# An online decision tree for vaccine efficacy trial design during infectious disease epidemics: The InterVax-Tool

**DOI:** 10.1016/j.vaccine.2019.06.019

**Published:** 2019-07-18

**Authors:** Steven E. Bellan, Rosalind M. Eggo, Pierre-Stéphane Gsell, Adam J. Kucharski, Natalie E. Dean, Richard Donohue, Matt Zook, W. John Edmunds, Frank Odhiambo, Ira M. Longini, Marc Brisson, Barbara E. Mahon, Ana Maria Henao-Restrepo

**Affiliations:** a Department of Epidemiology and Biostatistics, University of Georgia, Athens, GA, USA; b Center for Ecology of Infectious Diseases, University of Georgia, Athens, GA, USA; c Department of Infectious Disease Epidemiology, London School of Hygiene & Tropical Medicine, London, UK; d World Health Organization, Geneva, Switzerland; e Department of Biostatistics, University of Florida, Gainesville, FL, USA; f Department of Geography, University of Kentucky, Lexington, KY, USA; g Kenya Medical Research Institute, Centre for Global Health Research, Kisumu, Kenya; h Centre de recherche du CHU de Québec, Québec, Canada; i Département de médecine sociale et préventive, Université Laval, Québec, Canada; j Department of Infectious Disease Epidemiology, Imperial College, London, UK; k Centers for Disease Control and Prevention, Atlanta, Georgia, USA

**Keywords:** Vaccine trial design, Outbreaks, Epidemics, Vaccines, Public Health Emergency, Emerging infectious diseases, Scientific communication, Phase III trial, Decision support system, EVD, Ebola virus disease, PHE, Public Health Emergency, ZIKV, Zika virus, DENV, Dengue virus

## Abstract

**Background:**

Licensed vaccines are urgently needed for emerging infectious diseases, but the nature of these epidemics causes challenges for the design of phase III trials to evaluate vaccine efficacy. Designing and executing rigorous, fast, and ethical, vaccine efficacy trials is difficult, and the decisions and limitations in the design of these trials encompass epidemiological, logistical, regulatory, statistical, and ethical dimensions.

**Results:**

Trial design decisions are complex and interrelated, but current guidance documents do not lend themselves to efficient decision-making. We created InterVax-Tool (http://vaxeval.com), an online, interactive decision-support tool, to help diverse stakeholders navigate the decisions in the design of phase III vaccine trials.

InterVax-Tool offers high-level visual and interactive assistance through a set of four decision trees, guiding users through selection of the: (1) Primary Endpoint, (2) Target Population, (3) Randomization Scheme, and, (4) Comparator. We provide guidance on how key considerations – grouped as Epidemiological, Vaccine-related, Infrastructural, or Sociocultural – inform each decision in the trial design process.

**Conclusions:**

InterVax-Tool facilitates structured, transparent, and collaborative discussion of trial design, while recording the decision-making process. Users can save and share their decisions, which is useful both for comparing proposed trial designs, and for justifying particular design choices.

Here, we describe the goals and features of InterVax-Tool as well as its application to the design of a Zika vaccine efficacy trial.

## Highlights

• Phase 3 vaccine efficacy trial design during outbreaks and emergencies is challenging.
• InterVax-Tool (vaxeval.com) is a structured decision-support tool for trial design.
• Optimal design must include epidemiological, statistical, ethical, and logistical difficulties.
• Navigating these issues in real-time requires tools to assist in decision-making.
• Dynamic guidance, note taking, and tailored choices are key to good user engagement.

## 1 Background

Outbreaks or epidemics of emerging pathogens can pose a major threat to public health and may lead to public health emergencies (PHEs) [1]. Outbreaks can be unpredictable and often accelerate quickly, as seen during the recent 2014–2016 Ebola virus disease (EVD) and 2015–2016 Zika virus (ZIKV) epidemics. Preparedness activities are needed to increase the effectiveness and speed of epidemic responses, including clinical research [2], [3]. In particular, given the potential for safe and effective vaccines to control or prevent future outbreaks, it is imperative that the international community become better prepared to develop and test vaccines rapidly [2]. Even when candidate vaccines are available in the early stages of an emerging outbreak, as was the case during the 2014–2016 EVD epidemic, vaccine efficacy evaluation in phase III trials must be planned and executed quickly to obtain the most information possible. Design of such trials requires identification and recruitment of participants at risk of infection; engagement with local communities and national and international regulatory authorities; and consideration of a trial design’s ethicality, feasibility, and acceptability [4].

Improving the design of vaccine efficacy trials can be achieved by advance consideration of challenges that might arise in the context of a PHE, and establishing how these challenges affect key choices about trial design [5], [6], [7], [8], [9]. These challenges depend on factors such as the characteristics of the pathogen itself, the candidate vaccine under study, local health systems infrastructure and laboratory capacity, and the sociocultural context of the affected area(s). Multi-faceted and interconnected considerations are complex, and decision support tools that help clarify key issues can play an important role in aiding the trial design process.

Navigating these considerations during emerging outbreaks requires effective decision making because epidemic dynamics can rapidly change. In particular, this difficult environment requires clear understanding and communication of how decisions on trial design affect downstream choices. Decision support systems have previously been developed to support clinical decision-making [10], and to guide implementation of epidemic interventions [11], [12]. However, no decision support system has been developed to assist in the design of phase III vaccine trials during a PHE.

To address this issue, we have generated an interactive decision support tool for phase III vaccine trial design, InterVax-Tool (http://vaxeval.com). Through an interactive decision tree, which is a branching diagram showing choices, InterVax-Tool steps through key decision points, and demonstrates how particular characteristics (such as those described above) affect those decisions. InterVax-Tool is designed for use by diverse stakeholders, including national and international authorities, public health practitioners, policymakers, and others, and facilitates structured dialogue amongst stakeholders using a common framework.

## 2 Implementation

While substantial research exists on vaccine efficacy trial design [13], there is much less research on trial design for emerging infections, with the exception of novel work generated during the 2014–16 EVD outbreak [6], [14], [15], [16], [17]. Neither scientific literature nor public health agency guidance documents lend themselves well to urgent decision making: scientific literature is constantly changing and requires specialist knowledge for interpretation; and guidance documents from public health agencies, due to their lengthy and linear nature, can obscure the complex interdependencies between decisions [18]. InterVax-Tool forms part of work on the WHO Research & Development (R&D) Blueprint for preparedness for epidemics [3], [19] and was developed to harmonize with WHO preparedness tools by linking with newly developed guidance on trial design during outbreaks [4].

## 3 Structure

InterVax-Tool divides the design processes into *four decision topics*: (1) Primary Endpoint, (2) Target Population, (3) Randomization Scheme, and (4) Comparator; each has its own decision tree (Supplementary Information). Decisions within each of these topics are displayed as a hierarchy, giving a high-level view of the key choices and trade-offs. Users proceed through the decision topics, discussing and annotating each decision in turn.

**Fig. 1:**
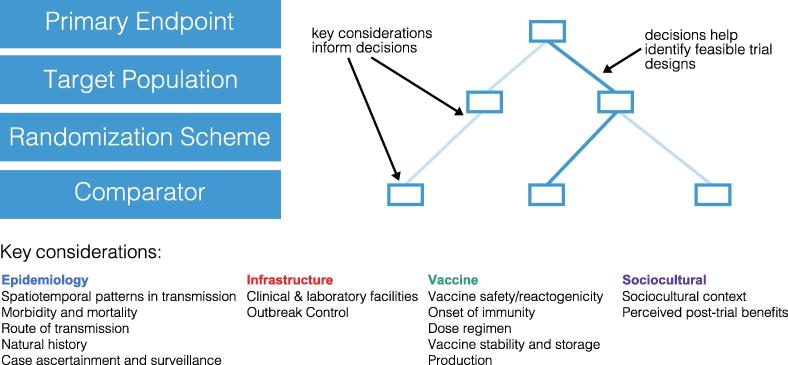
**Schematic of InterVax-Tool’s decision process.** Within each of four decision trees, users navigate a set of hierarchical decisions following guidance on how each of 14 key considerations affect the decision to pick one choice (blue rectangle) over another. During this process users take notes on the scenario under consideration as well as on their justifications for the decisions chosen through the four decision trees.

InterVax-Tool gives support to these decisions with content that describes *4 categories* of *key considerations*: (1) Epidemiology: pathogen transmission and epidemiology; (2) Infrastructure: health systems infrastructure; (3) Vaccine: characteristics of the vaccine under test; and (4) Sociocultural: the sociocultural context of the trial (Fig. 1). InterVax-Tool interactively displays all *key considerations* that are relevant to each decision and how they may affect that decision (Supplementary Information). This guidance includes citations both to scientific literature as well as to policy guidance documents [4]. InterVax-Tool allows users to assess implications for trial design, to identify areas where more information is needed, and ultimately to make informed decisions on the trial design.

## 4 Four decision topics

### 4.1 Primary endpoint

The primary endpoint of a trial is the main result that is used to determine efficacy, and for which participants are randomised, and the trial powered. This decision topic allows the user to determine if an infection-related endpoint is chosen, and whether that endpoint is quantified by a laboratory test or by participants meeting a case definition, which could be general symptoms of disease (such as fever with rash), or a severe subset of the disease (for example, encephalitis). Laboratory confirmation is the most common primary endpoint for vaccine trials. Users of InterVax-Tool then choose how cases are ascertained from populations as either laboratory confirmation or by clinical symptoms, and the assay used for detection (e.g. PCR, antibody, etc.) if laboratory confirmation was selected.

### 4.2 Target population

The target population is the population or sub-population that is enrolled in a trial. Users of InterVax-Tool are assumed to have made decisions on where the trial will likely be held before using the tool. The next decision is on the risk target for enrollment, for example, are participants enrolled from the general population, or in a particular geographic sub-areas (e.g. living close to a water source), risk group (e.g. injecting drug users), or contact group (e.g. family of a case). The aim of choosing a target population is to maximize the statistical power of the trial, and to evaluate safety and efficacy in a representative group. The final decision in this tree is whether the trial is responsive during enrollment or not. Responsive trials identify target populations and recruit participants during the outbreak. Responsive designs can increase power by “following” the outbreak dynamics but are logistically challenging.

### 4.3 Randomization scheme

Randomization is the process by which participants are separated into those allocated the vaccine and those not. The first decision is either to randomize individuals or to randomize clusters of individuals to receive the vaccine. Further questions in this decision tree are only used if the user selects cluster randomization. The user must then choose between parallel and temporally-varying cluster randomized (stepped wedge) designs. Clusters may be stratified to allow risk-prioritized rollout of vaccination. And finally, within-cluster individual randomization (two-tiered randomization) can be selected.

### 4.4 Comparator

Events in the comparison group (often termed the “control group”) are compared with the intervention or “vaccine group” of the trial. Participants in the comparison group may be blinded to their intervention status, which means they do not know if they have received the vaccine or not. This can be done if the participants receive a sham vaccine or “placebo”. This vaccine can be an active control, which is a real vaccine, but with no known impact on the disease under study. If no placebo or active control is used, then the participants know if they receive the vaccine or not, and the design is an open-label trial. If blinding is used, if both the participants and trial researchers do not know the status of participants, the trial is double-blinded. Inverted single-blind can be used, where the participants know if they have received the active vaccine but investigators do not. The final decision is if the comparison group receives the vaccine after a delay, or at the close of the trial (or never). For example, delayed comparison was used during the *Ebola ça Suffit* phase 3 vaccine trial in 2015–16 [20], [21].

## 5 Usability

**Fig. 2:**
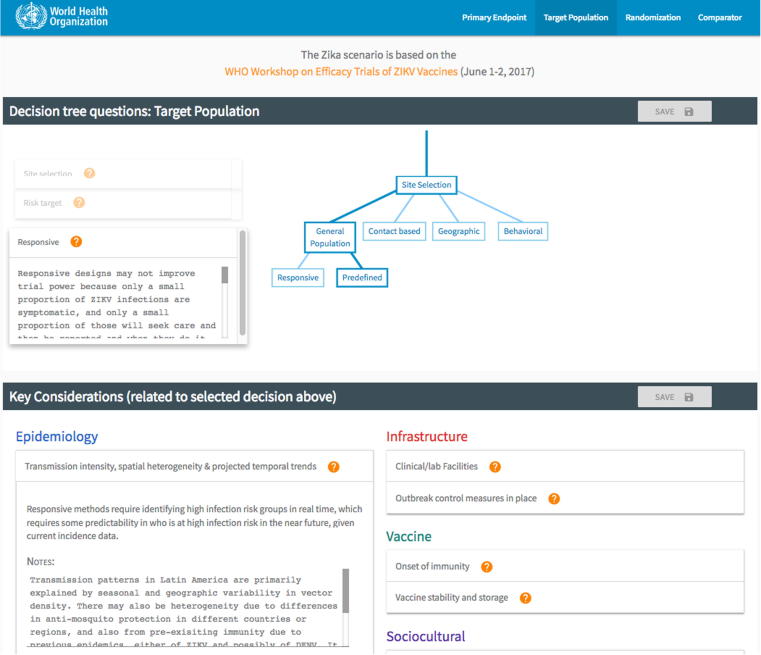
**Screenshot of InterVax-Tool at****http://vaxeval.com****.** The decision tree with decisions and notes is presented on the top portion. The lower portion provides content on how relevant key considerations impact the active decision in the tree and provides the opportunity for users to take notes on each key consideration, building a description of the scenario during the process.

InterVax-Tool facilitates open decision-making, accountability, and shareability by allowing users to take notes on their decisions and on how each key consideration applies. Notes made on decisions may reflect discussion about why a particular decision would be made or the tradeoffs between different choices (Fig. 2) and are anchored to that decision. Notes entered on key considerations are available at each decision point. For instance, if users record notes on a vaccine dose regimen while contemplating the primary endpoint, they will see and be able to update these same notes again when considering how dose regimen might impact the choice of comparator arm. This continuity in record-keeping generates a comprehensive record of the epidemic scenario under examination as well as justification for the best trial design(s). Scenarios and all annotation can be saved to an online database and loaded later, shared with others, or exported to a printable file.

InterVax-Tool works in settings with low bandwidth internet access, and promotes user engagement through an interactive user-friendly design [22]. It is completely free to use and open to all users online. Completed trees are stored in a database and retrieved using an email address and PIN code.

A five-minute video tutorial gives an introduction to the user interface. InterVax-Tool also contains a completed tree on ZIKV vaccine trial design scenarios. This tree, plus completed trees shared by other users will be an excellent resource when using InterVax-Tool for capacity building in vaccine trial design.

The WHO R&D Blueprint working group on phase 3 vaccine trials also generated a detailed guidance document on vaccine trial design [4] which is integrated with InterVax-Tool [23].

## 6 Case Study: Zika vaccine efficacy trial design

We piloted InterVax-Tool at the WHO Workshop on “Efficacy trials of ZIKV Vaccines: endpoints, trial design, site selection” in June 2017 to assist in the design of ZIKV vaccine efficacy trials [24]. At this meeting, a group of 30 experts used InterVax-Tool to discuss and refine options for potential phase III vaccine trials in Latin America. We displayed VaxEval.com on a projector, and used the site like a decision-making agenda, taking each decision point in turn. To lead the structured discussion we had a facilitator who was familiar with the website, key questions in trial design, and the scenario under study. The facilitator then prompted the key considerations for each decision to the group, and made sure these considerations were covered.

In future, we recommend a separate note-taker, who can input those live into the site during the discussion. At the end of each decision point, the facilitator should be sure to summarize and reach consensus, so that all participants are satisfied with the decision chosen and the information recorded into the site. Note that multiple trees can be used if needed, for example if some decisions lead to different preferred trial designs.

By using InterVax-Tool, the participants quickly focused their discussion on the three key areas where more information is needed to inform ZIKV vaccine trial planning: (1) the ability of serological assays to identify specific past flavivirus infections, i.e. determining serological positivity for ZIKV against a background of potential cross-reactivity from dengue virus (DENV; a related flavivirus); (2) forecasted or expected incidence required for feasible sample sizes; and (3) whether to use laboratory-confirmed ZIKV infection (whether asymptomatic or symptomatic) or laboratory-confirmed symptomatic Zika disease as the primary endpoint. The discussion quickly clarified that severe complications from ZIKV infection, such as Zika congenital syndrome or Guillain-Barré syndrome, were rare outcomes and, therefore, infeasible as primary endpoints. The ZIKV vaccine pipeline had 45 vaccine candidates at the time of the meeting, so participants agreed to delay focus on vaccine characteristics until candidate vaccines were approaching phase III trials. Meeting attendees agreed that the InterVax-Tool allowed a diverse group of experts to quickly narrow down the range of design possibilities and agree on important knowledge gaps that needed further attention.

## 7 Discussion

We created a web-based interactive decision tree tool, InterVax-Tool, to support decision making on vaccine trial design. By focusing users on the key decisions, trade-offs, and interdependencies, InterVax-Tool allows multidisciplinary users to quickly identify the key issues for each outbreak scenario and thereby assist the response to public health emergencies. It allows sharing and documentation of decision processes and provides scientific content to aid in the decision-making process.

InterVax-Tool is not intended to give a single answer on the optimal trial design for a given scenario, but rather to promote organized, efficient, and transparent discussion. In fact, the tool discourages the notion that there may be a single ideal design for any particular scenario and encourages participants to consider tradeoffs between multiple trial design decisions.

Additionally, a key component of vaccine trials is sample size calculations. The diversity and complexity of possible trial designs that can be generated using InterVax-Tool make generalizing about sample sizes very difficult, and we do not think this could be presented in a user-friendly way alongside the underlying decision tree process. Sample size calculations are discussed in the linked WHO Blueprint Working Group publication [4] and in textbooks on trial design [13]. We suggest that once feasible trial designs are identified, users of InterVax-Tool could consult with statisticians and epidemiologists to generate required sample sizes.

The guidance presented within InterVax-Tool reflects the collective expertise of this working group and its attempt to summarize the scientific literature on vaccine efficacy trial design. We encourage user feedback so that content can be updated to reflect the state-of-the-art on all facets of trial design.

Because InterVax-Tool is organized in a hierarchical decision tree framework, decisions are reflected as discrete choices between two or more options. In reality, some decisions may fall along a spectrum and not all categories are mutually exclusive. Nonetheless, the categorization scheme used encourages discourse and planning, though it simplifies some aspects of trial design.

Further, because InterVax-Tool aims to avoid user fatigue, some trial design options were excluded (e.g., factorial design and interim analysis options). The guidance within InterVax-Tool provides ample reference to the scientific literature on these topics as well as to the complementary, comprehensive WHO guidance document [4].

An important feature of the tool is the ability to transmit annotated decision trees and design justifications between users. For example, investigators could use the tool to provide their justification for a particular trial design to regional public health authorities. The tool may be similarly useful to vaccine manufacturers thinking about trial scenarios for a vaccine early in the pipeline. Finally, the tool may be useful in capacity building, providing students of trial design and emerging infections with a lens into this complex decision process. The conceptual design of this tool may be applicable to many other aspects of public health or other disciplines in which rapid, transparent, highly technical, and interdisciplinary decision making is necessary to address an urgent problem.

## 8 Conclusions

Intervax-Tool is decision-support tool for designing vaccine efficacy trials during emerging disease epidemics and outbreaks. It is web-based, interactive, and contains up-to-date scientific information to support real-time decision-making on trial design. Interactive tools are needed to demonstrate the complex interdependencies in trial design choices, and how many factors can affect trial feasibility.

## Funding

SEB was supported by National Institute of Health (NIH) National Institute of Allergy and Infectious Diseases grant K01AI125830. RME acknowledges funding from the National Institute for Health Research through the Health Protection Research Unit in Immunisation at the London School of Hygiene & Tropical Medicine in partnership with Public Health England, from an HDR UK Innovation Fellowship (grant MR/S003975/1), and from the Innovative Medicines Initiative 2 (IMI2) Joint Undertaking under grant agreement EBOVAC1 (grant 115854). The IMI2 is supported by the European Union Horizon 2020 Research and Innovation Programme and the European Federation of Pharmaceutical Industries and Associations. AJK was supported by a Sir Henry Dale Fellowship jointly funded by the Wellcome Trust and the Royal Society (grant 206250/Z/17/Z). NED and IML were supported by National Institutes of Health (NIH) grants U54 GM111274 and R37-AI032042, and WHO funding. WJE acknowledges VEEPED (Vaccine Efficacy Evaluation for Priority Emerging Diseases) for funding. VEEPED is funded by the National Institute for Health Research using Official Development Assistance (ODA) funding.

The views expressed are those of the authors and not necessarily those of the funders. The funders had no role in study design; in the writing of the report; or in the decision to submit the paper for publication. The findings and conclusions in this report are those of the authors and do not necessarily represent the official position of the US Centers for Disease Control and Prevention.

## Declaration of Competing Interest

The authors declare no competing interests.
