# Supplementary material for: An online decision tree for vaccine efficacy trial design during infectious disease epidemics: The InterVax-Tool
Source: Vaccine. 2019 Jul 18;37(31):4376–81. doi: 10.1016/j.vaccine.2019.06.019 (PMC6620503; doi:10.1016/j.vaccine.2019.06.019)
Supplement: Supplementary Data 1 [file mmc1.docx]

# Supplementary Information for InterVax-Tool

This supplement contains all information in the Intervax-Tool website <http://VaxEval.com> as of May 28 2019.

## Complete trees

The website dynamically displays available or unavailable decisions. Full trees are presented here for information.


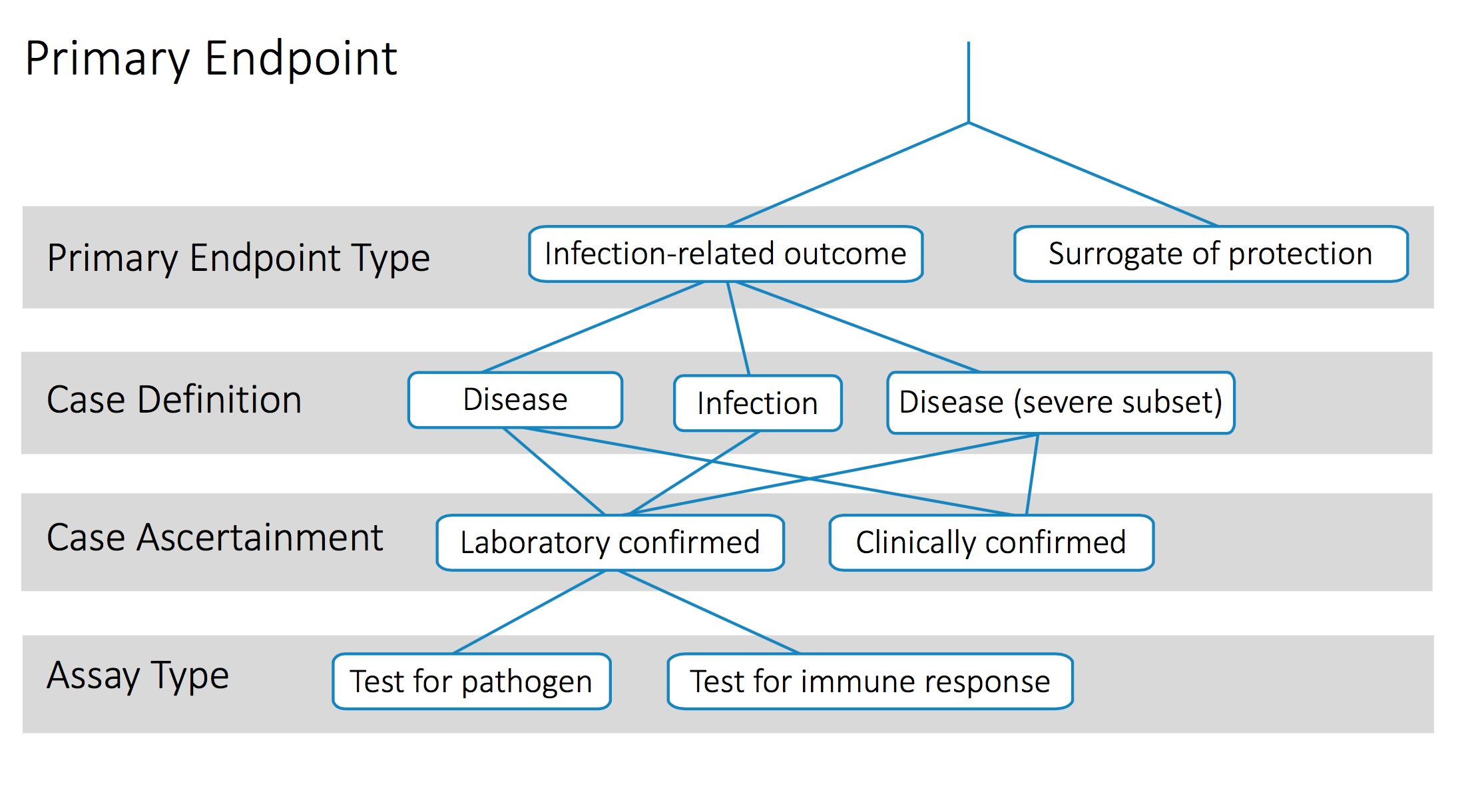


Figure 1. The complete tree for decisions on Primary Endpoint.


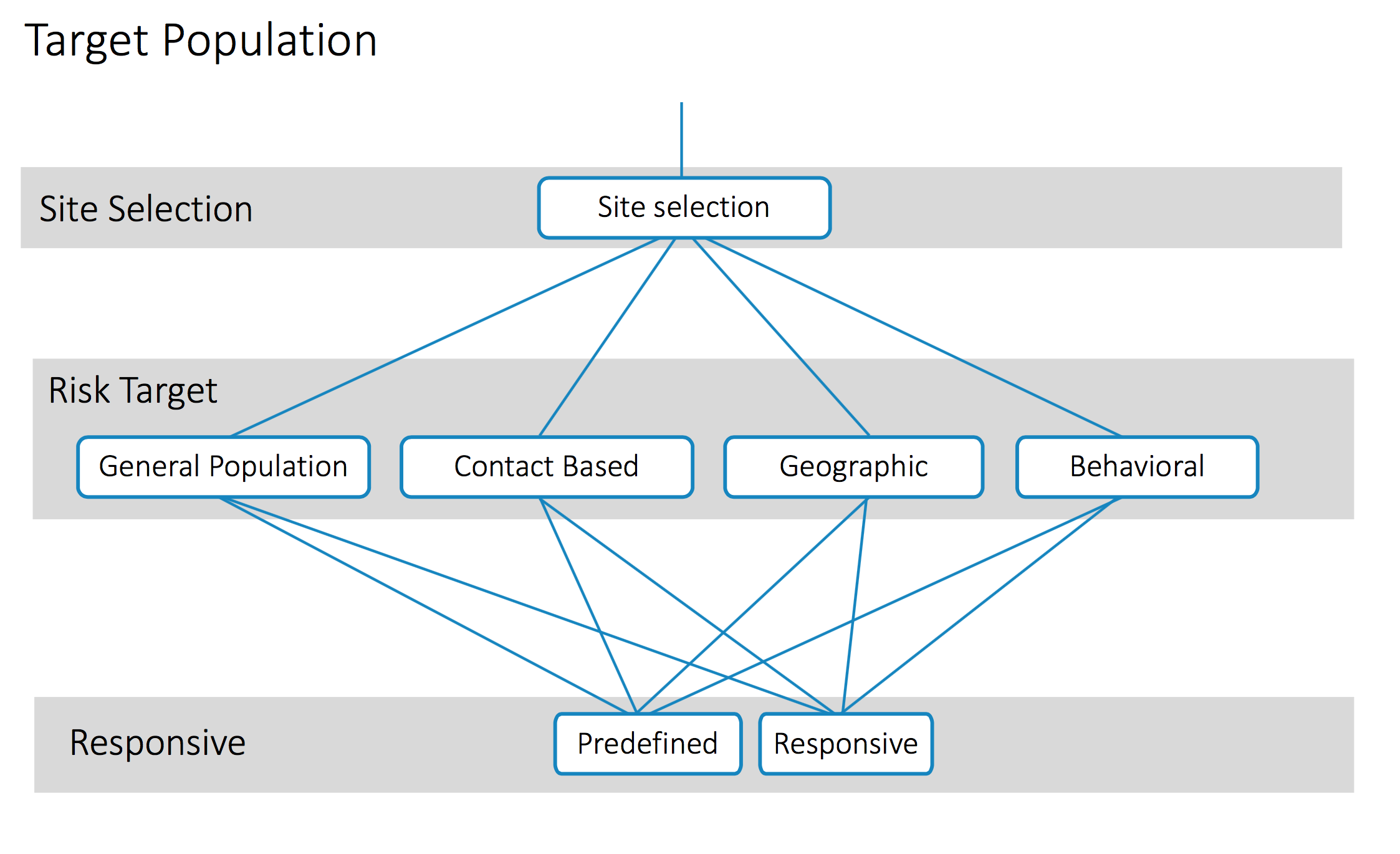


Figure 2. The complete tree for decisions on Target Population.


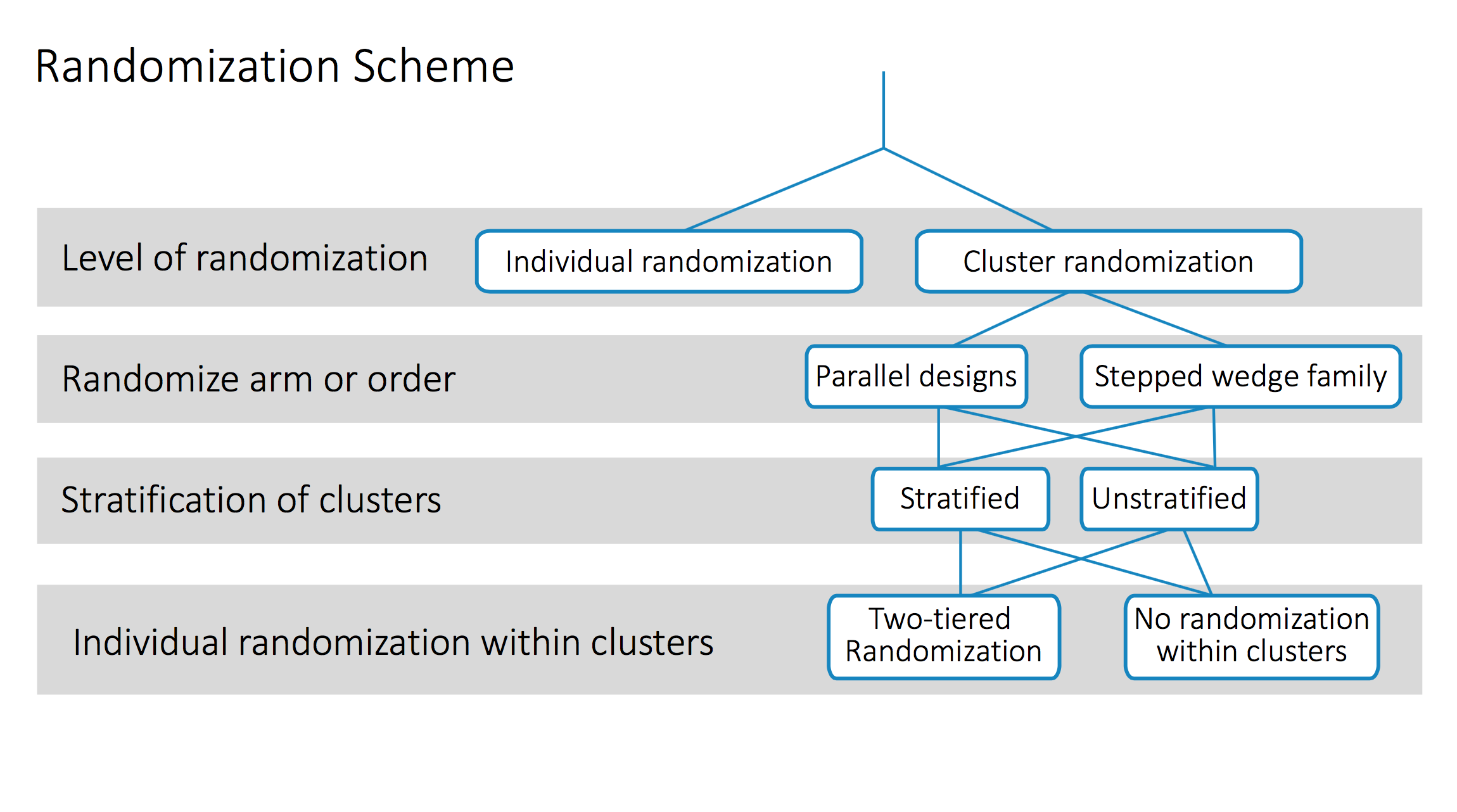


Figure 3. The complete tree for decisions on the Randomization Scheme.


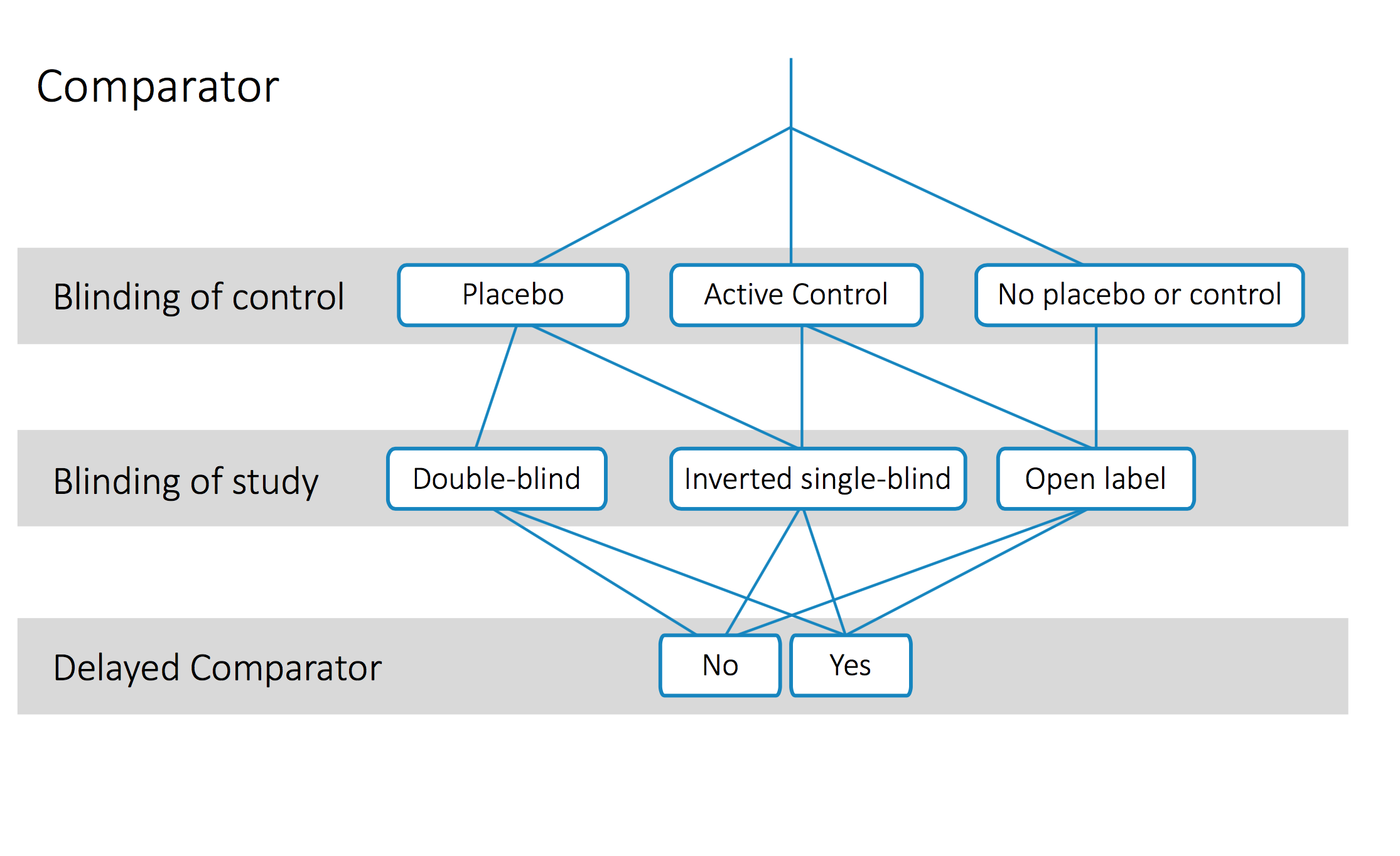


Figure 4. The complete tree for the decisions on Comparator.

## Definitions of decision points

| Decision Tree | Which question | Option | Definition |
| --- | --- | --- | --- |
| Endpoint | Primary endpoint type | Infection-related outcome | An outcome that is measured using a biological marker of infection. |
|  | Primary endpoint type | Surrogate of Protection | The end point is a biological marker that is thought to indicate immune protection. |
|  | Case definition | Disease (broadly defined) | Symptomatic changes in a participant that result from infection. Disease for all pathogens tends to be heterogeneous across individuals and care must be chosen to define a case definition that optimizes the balance between sensitivity, specificity, and choosing a target endpoint that reflects the target disease burden. |
|  | Case definition | Infection | Infection of the participant by the pathogen under study. |
|  | Case definition | Disease (a highly severe subset) | A particular grade of symptom state reached by a participant as a result of infection. |
|  | Case ascertainment | Laboratory-confirmed | Confirmation of infection by a test of a sample taken from the participant, e.g. PCR, antibody test, etc on blood or sputum sample. |
|  | Case ascertainment | Clinically-confirmed | Confirmation is made by a trained health professional without testing in a laboratory. |
|  | Assay type | Test for pathogen | Test looks specifically for evidence of the pathogen, e.g. PCR, virus isolation, culture. |
|  | Assay type | Test for immune response | Test looks for evidence of participants response to infection, e.g. antibody titration, immune markers. |
| Target Population | Site selection | Site Selection | Choose the predefined geographic site(s) (e.g., country, districts, or towns) in which the trial will be conducted based on identification of areas in which individuals are (1) likely to be at a high infection risk during the trial, (2) likely to exhibit the primary endpoint upon infection, and (3) represent populations in which the vaccine will likely be rolled out if found efficacious.  The following choices of Target Population indicate choices made within predefined trial site(s), rather than the choice of predefined sites themselves. |
|  | Risk target | General Population | Within chosen sites, individuals are recruited without regard to whether they are contacts of known cases, live in specific geographic areas near recently identified cases, or perform roles or exhibit behaviors that increase infection risk. |
|  | Risk target | Contact-based | Contacts of cases as identified via contact tracing. |
|  | Risk target | Geographic | Geographic regions surrounding cases or hotspots of infection. This is differentiated from site selection by spatial scale--geographic-targeting of cases refers to higher spatial resolution (i.e. city blocks around a new case, or recruitment within specific neighborhoods within a city that exhibit high infection risk). |
|  | Risk target | Behavioral | Individuals are recruited based on characteristics that put them at high risk of infection, e.g. their occupation, their role, or a behavioural characteristic.  Responsive designs are not considered possible in this category because it is unlikely that new behaviors will be identified over the course of a trial that help identify individuals at high risk. On the other hand, the inclusion criteria for any design (e.g. responsive geographic or responsive contact designs) may focus on individuals exhibiting specific behaviors within those target populations. |
|  | Responsive | Responsive | During the trial new areas or individuals may become eligible for recruitment. Those areas or individuals are not known at the start of the trial. |
|  | Responsive | Predefined | Within chosen trial sites, all areas or participants who could join the trial are delineated at the start of the trial. |
| Randomisation | Cluster randomization or individual randomization | Individual | Randomization to trial arms occurs at the individual level such that the trial arm assignment of two individuals in the same area or group is independent. This contrasts with cluster randomization, in which entire groups of individuals are randomized to the same trial arm.  Stratification within individually randomized controlled trials should be considered when considerable heterogeneity exists across groups of individuals (e.g. stratifying on site). |
|  | Cluster randomization or individual randomization | Cluster | A group or region is randomised, and all individuals within that group or region are assigned to the same trial arm (e.g., vaccine or control). |
|  | Randomize cluster arm or order | Parallel Designs | All clusters (or strata or matched pairs thereof) are assigned to their randomized arm at the same time and remain in the same arm throughout the study (i.e., no cross-over). |
|  | Randomize cluster arm or order | Stepped wedge family | Vaccine is rolled out to clusters in a pre-defined random order such that clusters cross-over from unvaccinated to vaccinated at different time points throughout the study.  These designs will be incompatible with responsively driven designs because the the temporal timing of vaccination for a newly identified target population (i.e. as based on geography or contact networks) will likely be time sensitive such that randomizing vaccination order within groups of newly identified target clusters will be extremely inefficient. |
|  | Cluster stratification | Stratified | Randomisation decision includes information on each cluster to ensure balance of randomisation across clusters (e.g., randomisation might occur within rural/urban strata). |
|  | Cluster stratification | Unstratified | Randomisation decision does not include information on each cluster. |
|  | Individual randomization within clusters | Two-tiered randomisation | Within a cluster, individuals may then be allocated to receive vaccine or control. |
|  | Individual randomization within clusters | No randomisation within clusters | All individuals within a cluster receive either vaccine or control. |
| Comparator | Blinding of control | Placebo | A sham vaccine is used in control participants. |
|  | Blinding of control | Active Control | A different vaccine, unrelated to the infection under study, is used in control participants. For guidance on trials that compare more than one vaccine against the same pathogen, please see {AP1 document hyperlink}. |
|  | Blinding of control | No placebo or active control | No sham or alternative vaccine is used in control participants. |
|  | Blinding of study | Double blind | Neither the participant nor investigator knows if a participant is in the vaccine or control arm. |
|  | Blinding of study | Inverted single blind | The participant knows which arm of the trial they are in, but the investigator does not. |
|  | Blinding of study | Open Label | Both the participant and the investigator know the status of the participants. |
|  | Delayed comparator | No (delayed comparator) | The control arm does not receive the investigational vaccine during the trial. |
|  | Delayed comparator | Yes | The control arm receives the investigational vaccine after a delay. |

## Key Considerations

| Category | Consideration | Guidance |
| --- | --- | --- |
| Epidemiology | Transmission intensity, spatial heterogeneity & projected temporal trends | The transmission risk faced by potential trial subjects may vary between individuals by age, sex, family role, profession, and location. Historical transmission trends may be poorly or well-characterized (depending on data availability). Future trends may be projected with transmission models at varying levels of resolution, though usually with great uncertainty. When transmission heterogeneity is at least somewhat predictable, projections may help with choice of design and subject recruitment. Transmission between clusters within cluster-randomized trials can also cause contamination, which will dilute estimates of indirect or total protective efficacy. |
| Epidemiology | Morbidity and mortality (and demographic heterogeneity therein) | The urgency surrounding high case fatality rates or severe morbidity may stimulate discussion surrounding a trial's ethical acceptability. The case fatality rate may be highly variably across demographic groups but is less likely to be highly variable across regions except due to differential access to care or due to interacting cofactors. |
| Epidemiology | Route of transmission & demographic heterogeneity in exposure | Transmission may occur directly through respiratory aerosols or droplets, contact with bodily fluids, or sexually; via vectors; or from environmental exposure. Direct transmission routes that allow characterization of contact networks may aid in subject recruitment via risk-responsive designs. |
| Epidemiology | Natural history (latent, incubation & infectious periods) | When latent (duration between infection and onset of shedding) and incubation (duration between infection and onset of symptoms) periods are relatively synchronous, symptoms serve as a good proxy for shedding. This makes contact tracing more feasible in realtime and the resulting contact networks can be used in risk-responsive designs. In contrast, when shedding occurs before symptoms, contact tracing will lag behind the epidemic spread, impeding risk-responsive designs. However, particularly long infectious periods can make contacts of infectious cases clear targets for subjects when sufficient testing efforts make diagnosis likely to occur even before symptom onset (e.g. as in HIV). |
| Epidemiology | Case ascertainment and surveillance | Depending on symptom onset timing and ease of diagnosis, cases may be detected via either passive or active surveillance. The delay between infection and case ascertainment affects the speed of a trial as well as the feasibility of designs that rely on comparisons between groups receiving vaccine at different times. Laboratory confirmation and sensitivity and specificity of assays can affect power. |
| Infrastructure | Clinical/lab Facilities | Countries experiencing the outbreak may or may not have the facilities needed for the trial. This includes storage facilities for vaccines, labs (at the appropriate BSL level), testing facilities, trial staff, and appropriate clinical facilities, including intensive care units (ICUs), for trial participants. |
| Infrastructure | Outbreak control measures in place | The trial can leverage outbreak control measures that are already in place, such as contact tracing, surveillance and case ascertainment, or other interventions that provide useful infrastructure, staff, or linkages with communities. |
| Vaccine | Vaccine safety/reactogenicity | Risk of side effects from vaccination, including standard side effects, e.g. pain at injection site, transient fever, etc. Note that some normal reactogenicity symptoms can mimic the symptoms of the disease or infection under study. The study site should be equipped with health care facilities for adverse events. |
| Vaccine | Onset of immunity | The timescale over which an individual acquires protective immunity to infection/disease/transmission following vaccination. Note multiple-vaccine regimes may have different immune ramp up periods and that immune ramp up may be heterogeneous across individuals. |
| Vaccine | Dose regimen | Single dose or multi dose regimen and route of entry (vaccine intramuscular, subcutaneous, intradermal, oral, transdermal, etc.) |
| Vaccine | Vaccine stability and storage | Storage temperature, preservative needs, and reconstitution requirements of the vaccine. |
| Vaccine | Production | Consider how much vaccine is available for the trial, and how much can be produced in what time period. There may be variations in batches, and there may be unexpected delays with new vaccines. This is especially important for vaccines made on new platforms. |
| Sociocultural | Sociocultural context | Special attention to a country's level of development and the corresponding challenges associated with transportation and other logistics, and with establishing national and community-level buy-in.  Outbreak-affected countries may have limited or no experience with vaccine trials or other clinical trials, or limited or no capacity for reviewing protocols. Participants, health care workers and other stakeholders at the local or national level may have varying degrees of trust in domestic or international authorities, which can hamper buy-in. |
| Sociocultural | Perceived post-trial benefits of research | Special attention should be paid to situations when trial communities are unlikely to benefit from the results of the research. Expectations should be planned and managed before the trial starts. Post-trial rollout of a successful vaccine (if efficacious) may provide meaningful benefits to participants if they are expected to experience sustained infection risk after the trial. Capacity building or sustained infrastructure generated by the trial may also provide long lasting benefits to participants and help enhance buy-in and promote the principle of justice. |

## Guidance given on each decision tree

### Tree: Primary Endpoint

|  | Question 🡪 | Primary endpoint type | Case definition | Case ascertainment | Assay type |
| --- | --- | --- | --- | --- | --- |
|  | **Description** 🡪 | Choose the endpoint type. An infection-related outcome is strongly preferred when at all possible.  {Hyperlink: See WHO Guidance Document } | Because the end aim of vaccines is to prevent disease, disease-based case definitions are usually preferred. However, other case definitions may be preferable if disease is rare or occurs with substantial delay post-infection, severity is unpredictable, or in certain other situations (see below for further detail). | Laboratory-confirmation of cases should be done when possible to ensure high specificity of the endpoint outcomes. Case definitions based on clinical symptoms alone may be reasonable only when lab confirmation is impeded by insufficient infrastructure. | Testing for the pathogen directly is preferable because it is highly specific (i.e. low probability of false positives). Only in unusual cases (e.g. no reliable test, or very short-lived infection) should other tests be used. Serology-based tests are often challenging because of problems distinguishing between pathogen- and vaccine- derived immunity in vaccinated participants, which makes it difficult to ascertain cases in the vaccine arm. |
| **Cat.** | **Consideration** |  |  |  |  |
| Epi | Transmission intensity, spatial heterogeneity & projected temporal trends | If no transmission, surrogate is only option. Otherwise infection-related endpoints are extremely preferrable since there are many obstacles to using surrogate endpoints as conclusive evidence for vaccine efficacy {reference: Fleming and Powers 2012}{Hyperlink: See WHO Guidance Document 7.2} | When transmission is more rare or difficult to predict, endpoints with greater sensitivity should be chosen. These primary endpoints can be combined with secondary endpoints that increase specificity at the cost of sensitivity. | NA | Seroconversion to detect incident infection can be used as a primary endpoint for a rare disease, with active or passive symptomatic surveillance and laboratory confirmation of pathogen used as a secondary endpoint. |
| Epi | Morbidity and mortality (and demographic heterogeneity therein) | NA | If disease severity is variable and predictable based on demographic variables, then the endpoint should ideally reflect the clinical disease that it is hoped the vaccine will mitigate. Using infection as the primary endpoint may not reflect the vaccine's true ability to prevent disease if the vaccine does not stop infection but rather reduces disease morbidity conditional on infection. However, when the focal clinical disease occurs very rarely, disease-based definitions will have lower sensitivity for infection and reduce the statistical efficiency of detecting efficacy against infection. In such cases and if efficacy against infection is deemed sufficiently informative, active case-finding based on laboratory-confirmed infection endpoints may be optimal. {Hyperlink: See WHO Guidance Document 8.2.1.3} | If disease symptoms are not specific, then laboratory assay-confirmed infection (PCR or antigen-detection assays) or seroconversion assays may be appropriate endpoints. | NA |
| Epi | Route of transmission & demographic heterogeneity in exposure | NA | NA | NA | NA |
| Epi | Natural history (latent, incubation & infectious periods) | NA | For diseases with long incubation periods, endpoints may need to be based on infection instead of symptomatic surveillance. The proportion of infections that are symptomatic will also influence choice of infection versus disease as the endpoint (see morbidity and mortality). | NA | NA |
| Epi | Case ascertainment and surveillance | NA | NA | The sensitivity and specificity of diagnostic assays and clinical diagnosis algorithms will help determine their relative utility as endpoints. | Duration of time in which pathogen is detectable in the host affects the sensitivity of pathogen-detection assays. |
| Infra | Clinical/lab Facilities | Samples need to be shipped to lab to test surrogate of protection. Skills required for certain tests may be very specialised. {Hyperlink: See WHO Guidance Document 8.5.2} | Lack of nearby clinical facilities may challenge the use of symptomatic endpoints unless symptoms are particularly specific for the disease and recall bias is minimal for the condition. In such cases, cumulative incidence of self-reported disease at the end of the trial, accompanied with serological assays, could be used in place of real-time active surveillance of symptoms with laboratory confirmation. | Lack of nearby laboratory facilities may be problematic if biological samples are reliant on an adequate cold chain from the study site to a distant laboratory. Lack of local laboratory facilities can also slow down confirmation of cases, which could challenge trials that responsively recruit cases (see Target Population subtree). These challenges could be overcome by improving logistics to speed up sample transport and testing, by building local laboratories, or by relying on laboratory endpoints that can be collected all simultaneously at the end of the trial (i.e. serological assays) if sufficiently sensitive and specific assays are available. | Depends on availability of assays and local capacity to perform them. |
| Infra | Outbreak control measures in place | NA | Outbreak control measures that rely on active or passive surveillance can be leveraged if the trial endpoint matches case definitions used during outbreak control, or if such definitions can be used as a starting point from which to identify a subset of individuals in which to test with more specific assays for the trial endpoint. | NA | NA |
| Vacc | Vaccine safety/reactogenicity | NA | NA | NA | NA |
| Vacc | Onset of immunity | NA | NA | NA | NA |
| Vacc | Dose regimen | NA | NA | NA | NA |
| Vacc | Vaccine stability and storage | NA | NA | NA | NA |
| Vacc | Production | NA | If vaccine supply is sufficiently limited so as to constrain trial sample size, then more sensitive case definitions will be needed to ensure sufficient power. | NA | NA |
| Socio | Sociocultural context | Surrogates of protection usually require blood (or other) samples. Blood draws, and transportation of materials out of the study area must be informed by local practices. | The choice of endpoint should be perceived as acceptable by the affected population.  In addition, the feasibility of collecting certain endpoints should be informed by the clinical facilities available to participants, and patterns of healthcare seeking in the target population. | Lack of nearby laboratory facilities may be problematic if biological samples are reliant on an adequate cold chain from the study site to a distant laboratory. It can also slow down confirmation of cases, important in a reactive trial. In such cases, logistical constraints may need to be overcome, or reliance on endpoints that can be collected all simultaneously at the end of the trial (i.e. serological assays) may be more useful. | NA |
| Socio | Perceived post-trial benefits of research | NA | The measurement procedure for ascertaining the endpoint should be perceived as acceptable by trial participants. | NA | NA |

### Tree:Target Population

|  | **Question 🡪** | **Site selection** | **Risk target** | **Responsive** |
| --- | --- | --- | --- | --- |
| **Description🡪** | | Choose sites that increase the chance of observing your chosen endpoint and that reflect areas that would likely receive post-trial benefits from an effective vaccine. | Choose the target population that will maximize the chance of observing your chosen endpoint (i.e. a population that exhibits high infection risk and, conditional on infection, a high probability of exhibiting that endpoint). The choices here reflect decisions taken to maximize power. Related decisions regarding endpoint choice to maximize trial power can be found in the Primary Endpoint subtree. Investigators will also have to determine additional inclusion criteria based on safety concerns.  While presented as such, the choices here are not necessarily mutually exclusive. For instance, one could do a responsive trial that targets all individuals working with animals (behavioral) within city blocks surrounding incident cases (geograhic). | Choose whether, within each trial site, the target population will be identified prior to the start of the trial or whether the target population will be responsively identified based on surveillance conducted throughout the duration of the trial to increase the chance that high infection risk individuals are enrolled. |
| **Cat.** | **Consideration** |  |  |  |
| Epi | Transmission intensity, spatial heterogeneity & projected temporal trends | Sites with ongoing transmission, especially those that are not yet in the declining phase of an epidemic would be ideal sites. For a discussion of challenges associated with trials conducted during waning epidemics. {Hyperlink: see WHO Guidance Document 10.1.1} | Target population should be chosen to maximize a trial's statistical efficiency by including individuals with high infection risk or with high projected infection risk. The breadth of the target population may need to be greater when infections are particularly rare or infection risk is difficult to predict (such that each person's a priori risk is very low). Appropriate trial simulators and simulated projections may help predict trial efficiency.   Spatial heterogeneity and speed of epidemic spread will impact geographic-based approaches. {Hyperlink: WHO Guidance Document 8.3 and 10.1.1} | Responsive methods require identifying high infection risk groups in real time, which requires some predictability in who is at high infection risk in the near future, given current incidence data. |
| Epi | Morbidity and mortality (and demographic heterogeneity therein) | Sites should be chosen to allow efficacy assessment in areas that contain individuals at the more severe end of the disease spectrum. | Target population should be chosen to allow efficacy assessment in individuals at the more severe end of the disease spectrum. This will help ensure that such populations will actually benefit if the trial is successful, particularly if the vaccine will only be used in such populations (i.e. versus widespread vaccination to create herd immunity). If severe disease is rare or difficult to predict based on demographic variables, then it will be difficult to choose the target population to specifically include those with severe disease. However, it should be kept in mind that the high risk population chosen should be similar enough to those who would be vaccinated post-trial such that efficacy results from the would be sufficiently generalizable {reference: Hayes and Moulton 2008:38)} {Hyperlink: See WHO Guidance Document 8.3} | NA |
| Epi | Route of transmission & demographic heterogeneity in exposure | NA | Target population should reflect known patterns in the route of transmission. For instance, individuals with risky sexual behaviors could be targeted for trials of vaccines against sexually transmitted infections; similarly, individuals inhabiting optimal vector habitat could be targeted for vaccines against vector-borne diseases. For some directly transmitted pathogens, it may be feasible to target case contacts for inclusion (i.e. risk-responsive designs).  The route of transmission will affect the utility of some of these targeting criteria, e.g. waterborne infection may render contact-based criteria ineffective at recruiting high risk participants. | Routes of transmission that are easily characterized (sexual transmission, direct transmission, localized vector/environmental transmission) facilitate the use of responsive designs based on contacts, geography, or behavior. In contrast, it may be challenging to develop responsive trial designs for pathogens for which transmission routes have been poorly characterized. {Hyperlink:See WHO Guidance Document 8.5.1} |
| Epi | Natural history (latent, incubation & infectious periods) | NA | Choice of target population should reflect how fast contacts would need to be identified, enrolled and vaccinated for the protective effect to occur early enough to prevent transmission (see onset of immunity). {Hyperlink:See WHO Guidance Document 8.5.1} | Slower natural histories increase the feasibility of responsive designs because they allow more time to determine where the epidemic is spreading and to identify potentially high infection risk individuals. Natural histories in which symptom onset coincides or predates the onset of shedding also increase the feasibility of responsive designs because they allow the trial to responsively target participants before they are exposed to an infected (but not yet infectious) individual. {Hyperlink:See WHO Guidance Document 8.5.1} |
| Epi | Case ascertainment and surveillance | Sites with ongoing surveillance infrastructure may help facilitate case ascertainment within the trial. | If poor, high risk individuals may be hard to identify. | Case ascertainment and surveillance are critical to responsive designs. The success of such designs depend on the feasibility of finding cases early enough to identify their contacts or areas around them in which individuals will be at high future infection risk.  Designs that responsively identify individuals based on behavior (i.e. frontline caregivers during Ebola) will not necessarily depend as intensively on case ascertainment and surveillance because they are only responsive in the sense that new individuals may enter specific behaviorally-defined groups (e.g., new burial workers are hired during an epidemic). |
| Infra | Clinical/lab Facilities | Sites with adequate clinical and laboratory infrastructure may help facilitate case ascertainment and laboratory confirmation of cases. | The number of samples that need to be processed may vary for different target populations and based on trial sample size. Available lab capacity can impact trial feasibility. | Collection and movement of samples in multiple locations may be difficult in some contexts, e.g. where roads are poor, or where disruption is occurring in some regions. Some responsive designs may generate clinical samples in multiple regions or countries, potentially requiring dispersed clinical/lab infrastructure (depending on ease of transportation). |
| Infra | Outbreak control measures in place | NA | The vaccine trial must work in concert with outbreak control measures, and effort must be made so that it is clear what aspects of intervention are experimental and which are proven. | Outbreak control measures may be critical in determining if the risk group identified at the time of the trial design is still at risk at the time of implementation. High-risk groups can change as the epidemic proceeds, and due to the outbreak control measures that are already being implemented. |
| Vacc | Vaccine safety/reactogenicity | NA | Candidate vaccines with major safety concerns from Phase I-II trials should only be targeted to high risk individuals since the anticipated risks may outweigh the benefits in the general population.  The safety profile of the vaccine may mirror symptoms of infection, e.g. fever. Enhanced monitoring of participants when they are high-risk is necessary. Note, that if their occupation is what makes them high-risk, they may no longer want to do that occupation. | NA |
| Vacc | Onset of immunity | NA | How fast do you need to get to contacts for vaccine to work (see immune ramp up) | If the dose regimen is long in relation to latent period, this may challenge responsive designs. By the time individuals identified responsively as being high risk have comleted their regimen, they may either already have been infected, or their infection risk may no longer be elevated. |
| Vacc | Dose regimen | NA | Some designs, e.g. geographic & contact, may provide logistic drawbacks if you have to re-contact individuals for multiple doses. | NA |
| Vacc | Vaccine stability and storage | NA | Trials selecting risk groups with a low risk of the primary endpoint will require large trial population sizes. This means that large quantities of doses would be required and consideration must be made for the consequent stability and storage requirements needed relative to the availability of storagae and transport cabillities at trial sites.  Any constraints on vaccine transport may challenge designs that require rapid vaccine distribution to remote areas. Experimental vaccines also tend to be less shelf-stable (since they are in development). Some of these problems were solved in the Ebola Ça Suffit ring vaccination trial by using transport canisters, although these may not be appropriate for long term storage. | Responsive designs require rapid distribution of vaccine to potentially remote areas, which may require even more careful consideration of storage and transport requirements and capabilities. |
| Vacc | Production | Sites with good transportation infrastructure may be preferrable for vaccines that require a cold chain. | General population studies will usually require greater sample size and thus greater dose availability. | NA |
| Socio | Sociocultural context | Sites in which vaccine trials have been conducted before may have relevant local expertise and familiarity that will ease the execution of a trial. However, this should be balanced with an assessment of where the vaccine is needed most and considerations of equitable access to experimental interventions. | The selection of target population for the trial must be culturally and socially appropriate in the country holding the trial. There could be differences between countries or regions in the likelihood of some target groups enrolling in the trial, especially where there is perceived to be national or international involvement in the trial. Lack of trust and knowledge of vaccine trials may impact feasibility of certain types of trials, where some designs may not be acceptable politically, or may not be feasible due to lack of trust in established structures. | It can be more difficult to do responsive designs if there are poor transport links, or poor health infrastructure (see Clinical/Lab Facilities). Responsive designs may also require sensitisation and community engagement in many different geographic or cultural areas, often under time pressure. When trial participants come from many areas and many sociocultural and socioeconomic backgrounds, the level of trust in existing authorities may differ widely. Care should be taken to survey these variations with extra effort made to achieve buy-in from specific groups as needed. Care must be taken to ensure that even when enrollment happens quickly, adequate consent and buy-in is obtained. |
| Socio | Perceived post-trial benefits of research | Sites selection should consider how the population at a site would receive future benefit from the vaccine if shown successful. | Lack of trust and knowledge of vaccine trials may impact feasibility of certain types of trials, where some designs may not be acceptable politically, or may not be feasible due to lack of trust in established structures.It should be clear who will benefit either via post-trial vaccination (if the trial is successful) or via the use of infrastructure or capacity generated by the trial.  Choice of criteria will determine the diversity of geography and demographic makeup of the trial population. When trial participants come from many areas and many sociocultural and socioeconomic backgrounds, the level of trust in existing authorities may differ widely. Care should be taken to survey these variations with extra effort made to achieve buy-in from specific groups as needed.   Role-based criteria target individuals whose roles put them at a long term sustained risk of infection. Thus, individuals in those same roles are likely to benefit from vaccine rollout post-trial as well (e.g. health care workers) either directly as a result of vaccination, or indirectly via herd immunity. This also may be true for geography-based targeting (if the pathogen is likely to remain a risk in the same geographic area for a long time) but may be less true for contact-based criteria unless the infectious period of the disease is very long. | In responsive designs there is an extra urgency involved in developing community and participant buy-in because target populations may need to be enrolled shortly after their identification. Care must be taken to ensure that even when enrollment happens quickly, adequate consent and buy-in is obtained. |

### Tree: Randomisation

|  | Question 🡪 | Cluster randomization or individual randomization | Randomize cluster arm or order | Cluster stratification | Individual randomization within clusters |
| --- | --- | --- | --- | --- | --- |
|  | **Description** 🡪 | Individually randomized designs are far more efficient than cluster-randomized designs at demonstrating efficacy, i.e. they can achieve the same statistical power with a smaller sample size. The two reasons why a cluster-randomized design might in some cases be preferable are  (1) they are operationally and administratively easier to execute, which may be in rare situations be an important factor (2) cluster-randomization allows estimation of indirect vaccine effects (i.e. herd immunity), which may be important to measure for in certain scenarios (e.g. for transmission blocking vaccines). | Parallel designs are operationally simpler than designs in which the order of cluster vaccination is randomized (stepped-wedge family designs). The latter may also suffer from poor power if temporal patterns in infection risk are highly variable. | Stratification of clusters allows risk-prioritized rollout (i.e. clusters with higher infection risk receive vaccine first) whilst maintaining the rigor of randomization. This can increase power. | Two-tiered randomization allows estimating of both direct and indirect vaccine effects. This may be important information in some circumstances though indirect effects are usually not given as much consideration in application for licensure. Two-tiered randomization is, however, less statistically efficient (lower power per unit sample size) at measuring direct effects than an individually randomized controlled trial, and less efficient at measuring total effects than either a cluster-randomized trial. Two-tiered randomization may, however, be more statistically efficient at measuring direct effects than a cluster-randomized controlled trial, depending on the proportion of individual variation that is due to between-cluster variation (i.e., the interclass correlation coefficient). {Hyperlink: See WHO Guidance Document 9.1.4.1} |
| Cat. | **Consideration** |  |  |  |  |
| Epi | Transmission intensity, spatial heterogeneity & projected temporal trends | Vaccine licensure generally requires demonstration of efficacy (a direct protective effect) to individuals. For measuring efficacy, cluster randomization requires a bigger sample size than individual randomization. This is both because individuals within clusters are similar and because, due to transmission, their disease outcomes are dependent. Thus, cluster randomization should be avoided when efficiency is of great concern, e.g. when cases are rare or when it is difficult to predict in which populations they will occur.   An exception to this rule may occur when most transmission in clusters arises from within the cluster and when R0 is close to 1. In such situations, cluster randomization may be similarly statistically efficient. This is because, if individually randomizing and vaccinating part of each cluster, transmission may be eliminated in all clusters, leaving negligible cases in all clusters. Cluster randomization of the same trial population, in contrast, would leave some clusters entirely unvaccinated allowing for transmission chains therein and, given efficacy, reveal discrepant incidence between vaccinated and unvaccinated clusters  Evaluation of indirect protective effects (herd immunity) is usually a secondary goal. Cluster, but not individual, randomization can be used evaluate indirect effects. In particular, when indirect effects are expected to be the dominant effect (e.g. such as in transmission-blocking vaccines), then cluster-randomized trials may be the only viable means to provide a realistic estimate of the vaccine's public health benefit.  In cluster randomized designs, potential transmission between clusters can cause "contamination", whereby clusters in different arms interact such that the true vaccine effect is diluted. Strategies to reduce contamination are described in Hayes and Moulton (2008, 58 – 64) and include the use of well-separated clusters or buffer zones. {Hyperlink: See WHO Guidance Document 9.1} | Designs that randomize cluster vaccination order (stepped wedge family designs) are particularly statistically inefficient when infection risk trends differ substantially across clusters. Furthermore, when risk varies dramatically between clusters, randomized order designs may lead to high infection risk clusters being randomized to late vaccination dates, which is suboptimal both from the perspective of trial power and participant outcomes (though this can be addressed in part by cluster stratification, see next decision). | For parallel designs, stratification of clusters based on anticipated infection risk can help achieve balance in infection risk between trial arms or aid in equitable distribution (e.g. urban/rural stratification). For randomized order designs, cluster stratification can allow for randomized order while still ensuring that the highest risk clusters are prioritized for earlier vaccination (e.g. by randomizing order within the four highest risk clusters, then the next four highest risk, etc.). | If individual-level transmission is extremely heterogeneous, two-tiered randomization may increase power to detect *direct vaccine effects* (i.e., efficacy). If transmission is low and homogenous, it may not be preferable to randomize within clusters for measuring direct effects but will still aid in measurement of indirect effects. |
| Epi | Morbidity and mortality (and demographic heterogeneity therein) | NA | Randomized order (stepped wedge family) designs in such settings are likely to be motivated by perspectives that highlight the importance of rolling out an experimental vaccine quickly even whilst in the process of evaluating whether it is protective. For experimental vaccines, this will likely only occur when disease severity is so high that the anticipated benefits of an experimental vaccine are expected to outweigh its anticipated risks, even accounting for the risk of side effects and realistic estimates of whether the vaccine is at all protective. When risk varies dramatically between clusters, however, randomized order designs may lead to high risk clusters not receiving vaccine as fast as could otherwise be possible and this may undermine some of the anticipated ethical advantages of a fast rollout. | NA | Individual randomization may be perceived as ethically problematic or unacceptable to trial communities when disease severity is particularly high. Individual randomization in such circumstances has nonetheless been considered acceptable by most national and international research ethics governing bodies. |
| Epi | Route of transmission & demographic heterogeneity in exposure | Certain routes of transmission lead to well-defined clusters in which transmission primarily arises from infections within clusters versus from outside the cluster (e.g. sexual transmission and sexual partners; direct transmission and households/schools). Indirect effects (herd immunity) are more easily detected in such scenarios because these effects, while not necessarily greater, may be more easily measured because they are concentrated within a clusters versus spread across individuals in other clusters (contamination) or outside the trial population. | Some will argue that withholding a potentially lifesaving intervention such as an experimental vaccine may be ethically challenging for individuals at known extremely high infection risk (e.g. close contacts). Randomization in such circumstances has nonetheless been considered acceptable by most national and international research ethics governing bodies. | In outbreak settings baseline trial population characteristics that may correlate with infection risk may be poorly understood, which favours individual randomization. When epidemic is reasonably predictable and baseline characteristics are known, stratification may be used to maximize balance between arms and increase statistical efficiency.  For cluster-randomized trials, stratification of clusters based on h also allows risk-prioritized rollout to clusters whilst maintaining the rigor of randomization because clusters can be randomized within strata. Otherwise, without stratification, any non-randomized ordering (e.g. such as risk prioritized ordering) of clusters' vaccination timing must be chosen before clusters are randomized to arms so that there is no possibility of temporal confounding. | See transmission intensity, spatial heterogeneity, and projected temporal trends.  When transmission clusters within tight-knit groups (households, close contacts) that are enrolled in an infection risk-responsive fashion, individual randomization may not be acceptable to trial population. |
| Epi | Natural history (latent, incubation & infectious periods) | NA | See case ascertainment and surveillance text | NA | NA |
| Epi | Case ascertainment and surveillance | For cluster-randomized designs, the utmost care must be taken to ensure equal surveillance effort across clusters. {Hyperlink: See WHO Guidance Document 9.1.2} | Randomized order designs may be particularly problematic if the delay between each cluster's vaccination exceeds the delay between infection and case ascertainment. This is particularly true when the latter is highly variable since this makes it different to distinguish whether individuals in each cluster were infected whilst unvaccinated or whilst protected. | NA | NA |
| Infra | Clinical/lab Facilities | NA | NA | NA | NA |
| Infra | Outbreak control measures in place | NA | Interaction between outbreak control during delay (how long does outbreak response take w/in cluster?) | If outbreak control measures differ systematically across groups of clusters, it would increase statistical power to stratify across this variation. | If outbreak control measures vary substantially across clusters, individual randomization within clusters will greatly increase the statistical power for detecting direct effects relative to a standard cluster-randomized design (but will still be less statistically efficient than a standard individually randomized controlled design). |
| Vacc | Vaccine safety/reactogenicity | NA | NA | NA | NA |
| Vacc | Onset of immunity | NA | Long and variable immune ramp up periods can challenge the interpretation and reduce the statistical efficiency of randomized order designs. This is because the immune ramp up period is usually excluded from analysis and, even if identifiably known, long immune ramp up periods will reduce the number of clusters available for comparison at any given point in time. | NA | NA |
| Vacc | Dose regimen | NA | Longer regimens will increase the immune ramp up period (see Onset of immunity). | NA | NA |
| Vacc | Vaccine stability and storage | NA | NA | NA | NA |
| Vacc | Production | Cluster randomization requires a greater sample size to achieve a set statistical power than individual randomization. Cluster randomization may therefore be problematic when vaccine doses are sufficiently limited. | Because randomized order designs are generally less statistically efficient in epidemic settings, parallel cluster designs may be preferable when vaccine supply is limited. | Cluster stratification makes a cluster-randomized design more efficient because you can roll out to high risk clusters first, which allows more power for fewer doses. | Because individual randomization is more statistically efficient for detecting direct effects (i.e., efficacy), it may be advantageous to use a two-tiered design relative to simple cluster-randomization when vaccine supplies are extremely limited but measurement of indirect effects is still deemed important. |
| Socio | Sociocultural context | Cluster (versus individual) randomization may be easier to explain to trial participants in areas where understanding of clinical research is limited, but caution must be paid to misinterpretations that perceive cluster allocations as driven by unequitable access rather than randomization. Cluster randomization may facilitate informed consent at the community level additional to individual informed consent. For most scenarios, it will be well worth the effort to communicate to participants the utility of individual randomization when cluster randomization initially appears more acceptable because of the former's far greater statistical efficiency. | Difficulties in transportation and logistics in developing regions may challenge randomized order designs that rely heavily on precise timing of vaccination rollout.  Randomized order designs may make trials more acceptable to local/national stakeholders because they resemble vaccine rollout. However, randomized cluster vaccination order when risk varies substantially between clusters may be perceived as an unequitable distribution of vaccine. This may be partially remedied by stratifying within randomized order designs. | Stratification on factors believed to predict cluster infection risk or characteristics that may be perceived as related to equitable access (urban/rural, socioeconomic status) may enhance cross-community buy-in of a cluster-randomized design by avoiding misinterpretation about which clusters do and do not receive vaccine. | If cluster randomization is chosen over individual randomization (first decision in this subtree) for logistical or acceptability reasons, the same disinclinations will likely apply to two-tiered randomization. |
| Socio | Perceived post-trial benefits of research | Individual randomization may be deemed more acceptable by the trial community when investigators sufficiently explain their commitment to making vaccine available to all control participants at the end of the trial if the vaccine is found efficacious. Because cluster-randomized designs are usually less statistically efficient, they will yield definitive results more slowly for a given sample size and thus control participants may receive vaccine (if it is found efficacious) later as compared when they would in individually randomized designs. | Randomized order designs may increase acceptability because all participants eventually receive vaccine. However, stakeholders should be aware that this presumes that trial participants are better off being vaccinated than not a priori, which may only be true for individuals at high infection risk of an extremely severe disease. | Stratification on cluster risk allows risk-prioritized vaccine rollout to clusters, which increases trial power and, consequently, the speed with which control groups will be vaccinated if the vaccine is demonstrated to be protective. | Because two-tiered randomization may be more statistically efficient than simple cluster-randomized designs for detecting direct effects (depending on the intraclass correlation coefficient), the former design may evaluate the vaccine more quickly, allowing vaccination of control groups more quickly if the vaccine is found to be protective. |

### Tree: Comparator

|  | **Question 🡪** | **Blinding of control** | **Blinding of study** | **Delayed comparator** |
| --- | --- | --- | --- | --- |
|  | **Description 🡪** | Blinding is strongly preferred, but choice of placebo or active control may be influenced by other factors including ethical considerations, community acceptability, or ease of blinding of the control as based on the vaccine's characteristics. | Double-blinding is ideal (both trial researchers and participants are blinded to participants' trial arm) because it reduces the potential for bias. Exceptions in which double-blinding may be challenging include situations in which there are strong operational constraints e.g. different syringes for active and control vaccinations. | Delayed vaccination designs reduce a design's statistical efficiency and increase operational complexity. However, in certain circumstances such designs may be considered more ethical or be more acceptable to communities because it reduces control participants' amount of unvaccinated person-time. The extra acceptability of this reduction rests on the assumption that participants are better of vaccinated than not a priori, which may only be true for participants at extremely high risk of infection that causes very severe disease with high probability. |
| **Cat.** | **Consideration** |  |  |  |
| Epi | Transmission intensity, spatial heterogeneity & projected temporal trends | NA | NA | Using a delayed comparator arm reduces a design's statistical efficiency because each control individual contributes less person-time at risk to the analysis. Thus, when incidence is low (cases are rare), delayed vaccine comparator arms should be avoided if possible.   Using a delayed vaccine control versus a standard (unvaccinated) control arm may be perceived as more ethical or acceptable because it limits the time spent unvaccinated by control participants. However, when the risk of infection to control participants declines substantially before the delayed vaccination would occur, this undermines the anticipated advantage. Thus, delayed comparators should be avoided when control participants are not expected to be at risk any longer by the time their delayed vaccination would occur. This is particularly true given that, if a vaccine is demonstrated to be efficacious, control individuals (if still at risk) would likely receive the vaccine in a post-trial rollout regardless of whether they are in a delayed vaccination or standard control arm. Delayed comparators only achieve their perceived advantages in scenarios in which control participants would receive the vaccine far earlier than they would if the vaccine is protective (i.e. a 6 month delayed comparator will not do much if a trial is expected to achieve results within 3 months upon which control participants would be vaccinated anyways). |
| Epi | Morbidity and mortality (and demographic heterogeneity therein) | NA | NA | Delayed comparator arms are likely to be motivated by extremely high morbidity or mortality, such that the anticipated benefit from an experimental (unproven) vaccine is greater than the anticipated risk, based on realistic expert opinion. |
| Epi | Route of transmission & demographic heterogeneity in exposure | If symptoms are very obvious and the design is clustered, then clusters could potentially be unblinded by an extremely effective vaccine because no cases will occur in the vaccine arms. | NA | NA |
| Epi | Natural history (latent, incubation & infectious periods) | NA | NA | When case ascertainment relies on symptoms, delay length for delayed vaccination comparator trials should account for the length and variability of the incubation period (see Case Ascertainment and Surveillance). |
| Epi | Case ascertainment and surveillance | NA | NA | If using a delayed control arm, the delay should be informed by the variability in the time lag between infection and case ascertainment (e.g. for symptomatic detection this would be the incubation period). When the time between infection and ascertainment is highly variable, large delays will be necessary to ensure that there are sufficient windows of time when cases ascertained in vaccine and delayed vaccine arm participants can be considered to have arisen while participants were protected and unprotected, respectively {reference: Dean et al. 2016} |
| Infra | Clinical/lab Facilities | NA | NA | NA |
| Infra | Outbreak control measures in place | NA | NA | NA |
| Vacc | Vaccine safety/reactogenicity | Safety can be more challenging to assess with an active control design. {Hyperlink: See WHO Guidance Document 8.4.2} | NA | Delayed vacination designs will be less preferable for vaccines with higher safety concerns. {Hyperlink: See WHO Guidance Document 8.6.2} |
| Vacc | Onset of immunity | NA | NA | If using a delayed control arm, the delay should be informed to the anticipated length and variability of the immune ramp up period (time from vaccination to protection, given efficacy). Delays should be longer than the immune ramp up period so that there is a time window during which vaccinated participants are likely fully protected and delayed arm participants are not yet vaccinated. A trial's power will be greatly affected by the length of this time window. {Hyperlink: See WHO Guidance Document 8.6.1} |
| Vacc | Dose regimen | Participant blinding is challenged by multiple dose regimens because of the extra logistical obstacles associated with giving control arm participants multiple doses of an active control or placebo. | Similar challenges apply as for participant blinding but knowledge of participant assignment can be limited to vaccine administrator and restricted from the rest of trial staff. | Dose regimens that are spaced out over long time periods increase the immune ramp up period and will require longer delays before the delayed comparator arm is vaccinated (see Onset of immunity). |
| Vacc | Vaccine stability and storage | Certain vaccine types may be challenging to blind to participants (e.g. lyophilized liquid vaccines). {Hyperlink: See WHO Guidance Document 8.6.3} | Similar challenges apply as for participant blinding but knowledge of participant assignment can be limited to vaccine administrator and restricted from the rest of trial staff. | NA |
| Vacc | Production | NA | NA | Because delayed vaccine control arms require more doses than standard control designs, delayed designs should be avoided when vaccine supply strains the sample size to levels at which statistical power is of concern. Delays may however allow more vaccine to be produced. {Hyperlink: See WHO Guidance Document 8.6.3} |
| Socio | Sociocultural context | Community engagement is critical to establish community acceptability of control arms, placebos, and blinding. | NA | When trust is low, a delayed comparator can give participants and communities a better sense of equitable access. Furthermore, vaccinating the control group after some delay increases the speed with which a vaccine is rolled out and may lead to greater community benefits through indirect protection, should the vaccine be effective. However, delayed vaccination reduces statistical efficiency and comes with challenges in analytic interpretation. Delayed comparators require extra logistical effort that may be amplified in settings where transportation and logistics are already challenging. |
| Socio | Perceived post-trial benefits of research | Randomized designs that rely on control participants can be more easily justified to control participants and local communities when a concrete commitment of post-trial resources is made (e.g. either rollout of vaccine if found efficacious, or other permanent infrastructure created during trial). | NA | Delayed comparators reduce the relative importance of post-trial vaccine rollout since control participants are ensured of vaccination regardless of (and possibly before) a definitive trial outcome. However, because delayed comparators reduce the trial power, the post-trial benefits to individuals outside of the trial will be delayed/diminished. |
